# Draft Genome Sequences of Four Virulent *Aeromonas hydrophila* Strains from Catfish Aquaculture

**DOI:** 10.1128/genomeA.00860-16

**Published:** 2016-08-18

**Authors:** Hasan C. Tekedar, Salih Kumru, Attila Karsi, Geoffrey C. Waldbieser, Tad Sonstegard, Steven G. Schroeder, Mark R. Liles, Matt J. Griffin, Mark L. Lawrence

**Affiliations:** a College of Veterinary Medicine, Mississippi State University, Mississippi State, Mississippi, USA; b Warmwater Aquaculture Research Unit, Agriculture Research Service, U.S. Department of Agriculture, Stoneville, Mississippi, USA; c Bovine Functional Genomics Laboratory, Agricultural Research Service, U.S. Department of Agriculture, Beltsville, Maryland, USA; d Department of Biological Sciences, Auburn University, Auburn, Alabama, USA

## Abstract

Since 2009, a clonal group of virulent *Aeromonas hydrophila* strains has been causing severe disease in the catfish aquaculture industry in the southeastern United States. Here, we report draft genomes of four *A. hydrophila* isolates from catfish aquaculture that represent this clonal group.

## GENOME ANNOUNCEMENT

*Aeromonas hydrophila* is a Gram-negative opportunistic pathogen that has been recovered from a wide variety of hosts, including mammals and aquatic organisms. Catfish aquaculture is an important industry in the southeast United States, and since 2009 a clonal group of virulent *A. hydrophila* (VAh) isolates has caused significant losses. For comparative analysis, our research group released the complete genome sequence of a VAh strain from septicemic catfish (ML09-119, GenBank accession no. NC_021290) (1) and two other *A. hydrophila* strains from diseased fish (AL06-06, NZ_CP010947; and TN97-08, NZ_LNUR01000001) (2, 3). Here, we report four draft genomes of VAh strains isolated from farm-raised catfish in 2009 and 2010 (strains AL10-121, AL09-79, ML09-121, and ML09-122). These genomes will be used to compare against our previously sequenced *A. hydrophila* genomes and other *Aeromonas* genomes to clarify the variation within the VAh group.

*A. hydrophila* AL10-121, AL09-79, ML09-121, and ML09-122 genomes were sequenced using an Illumina Genome Analyzer IIx. The total number of reads and fold genome coverages were as follows: 8,107,077 reads with 224× coverage (AL10-121); 7,613,846 reads with 206× coverage (AL09-79); 7,094,460 reads with 192× coverage (ML09-121); and 6,199,052 reads with 176× coverage (ML09-122). Adaptor trimming, contig creation, and quality control of sequence reads were conducted using CLC Workbench version 6.5.1 (CLC Bio) and Sequencher version 5.4 (Gene Codes Corporation). *De novo* assembly was performed by CLC Workbench version 6.5.1.

For annotation, the draft genomes were submitted to RAST (4) and NCBI’s Prokaryotic Genome Automatic Annotation Pipeline (PGAAP) (5). Features of the draft *A. hydrophila* genomes are summarized in Table 1. Results indicate that all draft genomes are quite similar in terms of genome size, GC content, coding gene number, and tRNA number.

**TABLE 1:** Summary of genome sequencing results in the present study

Strain	Isolation location	Genome size (bp)	No. of contigs	G+C content (%)	Predicted genes/protein- coding sequences	No. of tRNAs	Accession no.	GI no.
AL10-121	Alabama	4,969,906	13	60.90	4,461/4,307	95	LRRW00000000	1012455666
AL09-79	Alabama	4,967,857	13	60.90	4,455/4,300	93	LRRV00000000	1012457531
ML09-121	Mississippi	4,965,942	14	60.90	4,456/4,301	96	LRRX00000000	1012457651
ML09-122	Mississippi	4,969,986	19	60.90	4,457/4,302	92	LRRY00000000	1012462552

The average nucleotide identity (ANI) between each of the four VAh genomes and the complete genome of VAh strain ML09-119 was 99.99% (6). By comparison, the ANI between *A. hydrophila* strain AL06-06 and the four VAh draft genomes was 96.6%. Functional comparison of the annotated VAh draft genomes against *A. hydrophila* ML09-119 showed that the VAh strains share the same functional elements except a few metabolic genes in glycine and serine utilization, as well as cysteine, biotin, and molybdenum biosynthesis. On the other hand, the VAh strains all encode several pathways that are missing in other *A. hydrophila* strains such as AL06-06. For example, VAh strains carry an inositol catabolism pathway, phage elements, and an RTX toxin cluster that are missing in *A. hydrophila* strain AL06-06. In contrast to the AL06-06 genome, none of the VAh strain genomes carry plasmid.

### Accession number(s).

The draft genome sequences of *A. hydrophila* strains AL10-121, AL09-79, ML09-121, and ML09-122 have been deposited in GenBank, and their accession numbers are found in Table 1. The versions described in this paper are the first versions.
